# Parallel Mechanism Composed of Abdominal Cuticles and Muscles Simulates the Complex and Diverse Movements of Honey Bee (Apis mellifera L.) Abdomen

**DOI:** 10.1093/jisesa/ieaa075

**Published:** 2020-10-24

**Authors:** Youjian Liang, Kuilin Meng, Jieliang Zhao, Jing Ren, Siqin Ge, Shaoze Yan

**Affiliations:** 1 Division of Intelligent and Biomechanical Systems, State Key Laboratory of Tribology, Department of Mechanical Engineering, Tsinghua University, Beijing, P.R. China; 2 DFH Satellite Co., Ltd., Beijing, P.R. China; 3 Key Laboratory of Zoological Systematics and Evolution, Institute of Zoology, Chinese Academy of Sciences, Beijing, P.R. China

**Keywords:** honey bee, intersegmental structure, parallel mechanism, abdominal motion

## Abstract

The abdominal intersegmental structures allow insects, such as honey bees, dragonflies, butterflies, and drosophilae, to complete diverse behavioral movements. In order to reveal how the complex abdominal movements of these insects are produced, we use the honey bee (*Apis mellifera* L.) as a typical insect to study the relationship between intersegmental structures and abdominal motions. Microstructure observational experiments are performed by using the stereoscope and the scanning electron microscope. We find that a parallel mechanism, composed of abdominal cuticle and muscles between the adjacent segments, produces the complex and diverse movements of the honey bee abdomen. These properties regulate multiple behavioral activities such as waggle dance and flight attitude adjustment. The experimental results demonstrate that it is the joint efforts of the muscles and membranes that connected the adjacent cuticles together. The honey bee abdomen can be waggled, expanded, contracted, and flexed with the actions of the muscles. From the view point of mechanics, a parallel mechanism is evolved from the intersegmental connection structures of the honey bee abdomen. Here, we conduct a kinematic analysis of the parallel mechanism to simulate the intersegmental abdominal motions.

Seemingly ingenious structures of animal bodies always fill us with admiration (Burrows and Gregory 2013, Saito et al. 2017, Faber et al. 2018, Wu et al. 2018, Zhao et al. 2018). The abdominal intersegmental structures enable insects, such as honey bees (Slessor et al. 2005, Trhlin and Rajchard 2011), dragonflies (Miller 1962), butterflies (Senda et al. 2012), and drosophilae (Llopart et al. 2002), to complete variable behavioral activities. But how are the complex movements of insect abdomen achieved? Here, we use the honey bee (*Apis mellifera* L.) as a typical insect to study the relationship between intersegmental structures and abdominal motions.

In the honey bee dance language (von Frisch 1967, Dyer 2002), the forager bees recruit their nestmates to food sources by performing dances. The information encoded in the dance would be decoded by recruits to guide themselves directly to the destination. Today almost all biologists have been convinced that the description about dance language was correct (Gould 1975, Timothy 1995, Sherman and Visscher 2002, Riley et al. 2005). Notably, flight attitude adjustment was a key factor that could not be ignored in the process of foraging. The honey bees modulated their abdominal postures to reduce the aerodynamic drag based on the speed of a translating visual pattern (Tien et al. 2011, Jonathan et al. 2013). But visual information was not the only sense to control their flight. Taylor et al. (2013) established a model to predict the abdomen response based on the non-linear combinations of air speed and optic flow. In addition, swinging motion of the honey bee abdomen was used to dissipate residual flying energy while landing (Zhao et al. 2017a). Furthermore, Zhao et al. (2015, 2016) revealed the bending mechanism of honey bee abdomen and illustrated the importance of the folded intersegmental membrane (FIM) in honey bee abdominal motions. The discovery of the bending mechanism was helpful for us to understand the behavioral activities of honey bee such as mating. To further reveal the function of muscles on abdominal motions, Liang et al. (2019) studied the particular assembles format of muscles and cuticle, and found that kinematics of Stewart platform could explains three-dimensional movement between the sternum and tergum in the same abdominal segment of honeybee. Besides, we also found that there were a dozen of muscles between the adjacent segments, and bionic design of morphing nose cone for aerospace vehicle based on the deformable mechanism of honeybee abdomen a parallel-serial mechanism was performed (Liang et al. 2020). However, what has hitherto been lacking is a thorough analysis of the structures, especially from the perspective of mechanics to interpret the abdominal motions.

This work focuses on the problem of the equivalent mechanism of the adjacent cuticles and the muscles connected to them. We find a parallel mechanism in the intersegmental structures. Results show that it is the parallel multi-driver structure that helps the honey bee abdomen produce various behavioral activities, such as waggle dance, flight attitude adjustment, and sting. Furthermore, a motion principle of honey bee abdomen is proposed based on the kinematic analysis. This dexterous structure makes the multifarious activities of honey bee abdomen more flexible.

## Materials and Methods

### Experimental Animals

This work was performed on adult worker honey bees. Honey bees were kept in a 120- × 40- × 60-cm transparent cage at room temperature and fed with honey and sucrose water. To simulate a natural environment, several potted plants were placed in the cage and a fluorescent lamp was used to provide adequate lighting. All honey bee specimens were collected from a single hive maintained at the intelligent bio-mechanical laboratory of Tsinghua University, Beijing, China (40.00 N, 116.33 E). We confirmed that no specific permission was required for such locations and activities, and that field studies did not involve endangered or protected species.

### Sample Preparation

To remove chemical contamination of the abdomen, the honey bees were dissected with the help of the stereoscope (Stemi 508, Carl Zeiss AG, Oberkochen, Germany). Subsequently, the dissected honey bees were washed with 0.1 mol/L phosphate buffer (pH = 7.2) three times and fixed in a 2.5% glutaraldehyde solution for more than 4 h. Then, specimens were washed with 0.1 mol/L phosphate buffer (pH = 7.2) three times again, dehydrated in an ethanol series (70, 80, 85, 90, 100%, 15 min every time), soaked in Tert-butanol, and dried with critical point drier.

### Microstructure Observation Experiments

In a first step, intersegmental microstructures of honey bee abdomen were observed with the help of the stereoscope (Stemi 508, Carl Zeiss AG). The viscera were taken off. In a second step, the specimens were sputtered with gold after drying with critical point drier and observed by the scanning electron microscope (FEI Quanta 200, FEI Company, Eindhoven, Netherlands).

## Results

**Fig. 1.**
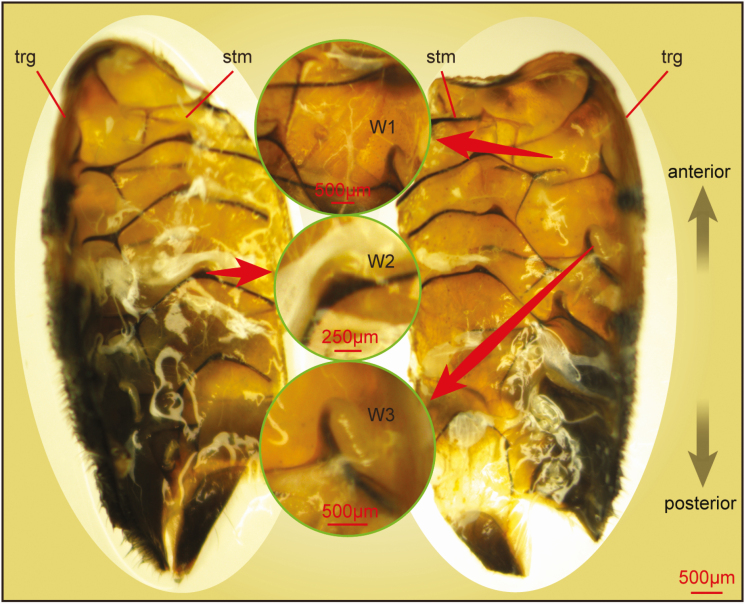
Internal microstructures of honey bee abdomen captured by the stereoscope. The abdominal body wall of honey bee is anatomized with the help of a stereoscope. The viscera are removed. There are some white gelatinous substances adhering to the terga and sterna. trg, tergum; stm, sternum; W, white gelatinous substance.

The body wall of honey bee abdomen was a crucial part of the honey bee to protect inner organs and tissues. To obtain the internal microstructures of honey bee abdomen, the cuticle was dissected with the stereoscope (Fig. 1). The viscera of honey bee abdomen were removed. The sterna and terga overlapped one another, each rear edge overlapping the front margin of the segment next behind. There were quite a few white gelatinous substances adhered to the terga and sterna. One end of W1 stuck to the upper edge of the tergum, and the other end of W1 stretched obliquely upward and located near the front margin of the anterior tergum. Besides, one side of W2 adhered to the sternum apodeme, and the other side of W2 may locate on the anterior sternum. In addition, W3 located between the apodeme of the tergal plate and the anterior tergum. The tergal plates and sternal plates could move along these elastic substances and carry out motions within a certain range. However, they could not be discerned exactly due to the limitation of magnification.

**Fig. 2.**
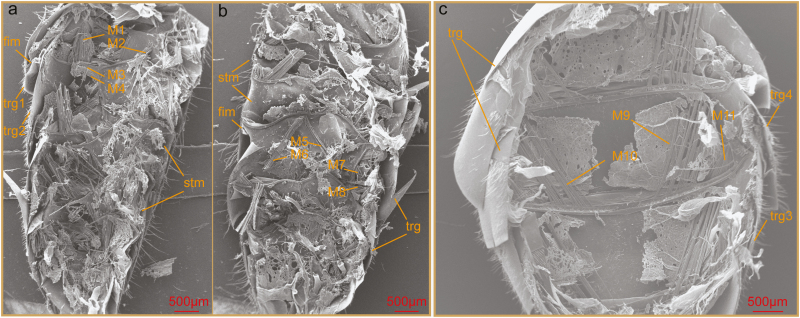
Intersegmental muscles and membranes of honey bee abdomen. (a) The left part (the left side of the longitudinal center axis while the terga are facing up) of the abdomen. The white gelatinous substances in Fig. 1 are muscles. A multitude of muscles and membranes exist between the terga and sterna. (b) The right part of honey bee abdomen. Two muscles, M3 and M4 are captured which are not detected in stereoscope observation. (c) The distribution of muscles on the terga of honey bee abdomen. trg, tergum; stm, sternum; M, muscle; FIM, folded intersegmental membrane.

In order to capture the intuitive aforementioned white gelatinous substances of honey bee abdomen, here we studied the inner microstructures by SEM. As shown in Fig. 2a, it was the left part of honey bee abdomen. Aforementioned white gelatinous substances represented muscles. The muscles M1 and M2 responded to the foregoing W1 and W2 in Fig. 1, respectively. Furthermore, the FIM started from the edge of the anterior tergum (trg1) and extended to the margin of the posterior tergum (trg2). It meant that the terga and sterna combined together through the muscles and FIM. The multifarious behavioral motions, such as expansion, contraction, and flexion of honey bee abdomen, were made possible by FIM. As manifested in Fig. 2b, it was the right part of honey bee abdomen. The muscles M3 and M4 located between the edge of the anterior sternal plate and the margin of the posterior sternum. The distribution of muscles on the terga was very neat and concise. As demonstrated in Fig. 2c, muscles M5 and M6 distributed symmetrically on both sides. Muscles M5 and M6 started from the edge of the posterior tergum (trg3) and located near the front margin of the anterior tergum (trg4). The muscle M7 responded to the foregoing W3 in Fig. 1. Results indicated that the muscles M1, M2, M3, M4, M5, and M7 were intersegmental muscles. It meant that there existed 12 bunches of muscles between the neighboring cuticles based on the symmetry of the muscles distribution.

The intersegmental muscles make a remarkable contribution to the honey bee abdominal motions. The muscle is equivalent to an actuator in engineering based on its function (Mill 1964, Wasserthal and Wasserthal 1980). The muscle can be considered as a prismatic pair, which is equivalent to a variable length limb (Liang et al. 2019). The junction between the muscle and plate can be regarded as a spherical pair.

Two adjacent cuticle segments are considered as the base platform and moving platform, respectively. The moving platform has 6 df relative to the base platform. Three correspond to rotational movement around the x-, y-, and z-axes, commonly termed pitch, yaw, and roll; df refer to the number of basic ways a rigid object can move through 3D space. There are 12 beams of muscles between the adjacent cuticles. If every muscle bundle is regarded as a variable length limb, the system composed of two adjacent cuticle segments and 12 intersegmental muscles will be a redundant input parallel mechanism including the base platform, the moving platform, and 12 variable length limbs. Thus, the number of limbs in this parallel mechanism can be reduced appropriately. However, only 6 of the 12 limbs can determine the position and orientation of the moving platform, and the other 6 driving limbs are redundant, which must be coordinated with the previous 6 driving limbs and cannot change their lengths independently. Therefore, from the perspective of determining the posture and movement of the bee’s abdominal cuticle segment relative to the previous one, it can be equivalent to a typical form of the parallel mechanisms, the Stewart platform (Stewart 1965, Dasgupta and Mruthyunjaya 2000), with six telescopic actuators connecting the base platform with the moving platform.

**Fig. 3.**
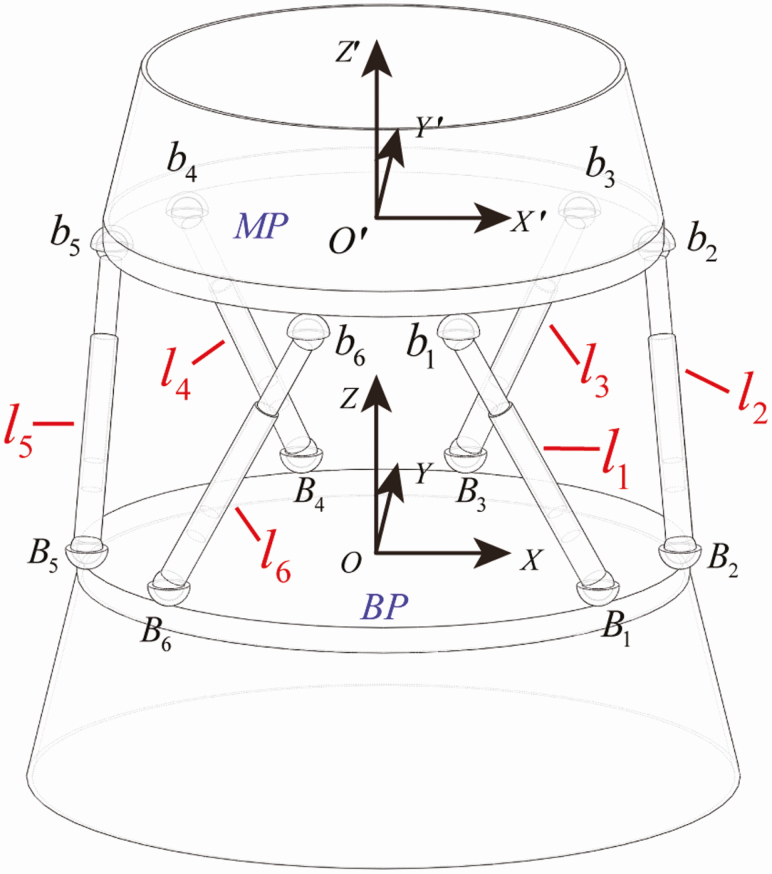
Schematic diagram of the parallel mechanism. The fixed coordinate system OXYZ is established on the base platform B1B2B3B4B5B6 and the moving coordinate system O'X'Y'Z' is built on the moving platform b1b2b3b4b5b6. The length of l1 is defined as the distance between B1 and b1. B1 and b1 are the center of the two spherical pairs, respectively. The rest limbs can be done in the same manner. MP, moving platform; BP, base platform; l, limb.

As shown in Fig. 3, it is the schematic diagram of parallel mechanism. The anterior cuticle is regarded as the moving platform and the posterior cuticle is perceived as the base platform. This parallel mechanism has six limbs, which are all composed of the spherical pair (S), prismatic pair (P), and spherical pair (S) in sequence. The fixed coordinate system *OXYZ* is built on the base platform *B*_1_*B*_2_*B*_3_*B*_4_*B*_5_*B*_6_ and the moving coordinate system *O′X′Y′Z′* is established on the moving platform *b*_1_*b*_2_*b*_3_*b*_4_*b*_5_*b*_6_. The vertical distance between the moving platform and the base platform is 600 mm. The radii of *b*_1_*b*_2_*b*_3_*b*_4_*b*_5_*b*_6_ and *B*_1_*B*_2_*B*_3_*B*_4_*B*_5_*B*_6_ are 534 and 584 mm, respectively. In the moving coordinate system *O′X′Y′Z′*, every vector ***R*′** can be transformed into the fixed coordinate system *OXYZ* by coordinate transformation.

$$R=[T]R^{\prime }+O^{\prime }$$ (1)

where [*T*] is the coordinate transformation matrix and ***O′*** is the origin of the moving platform. The expression of [*T*] is as follows while the moving platform rotating around x*-*axis:

$$\left[T_{\text{x}}\right]\text{=}\left[\begin{array}{ccc} 1 & 0 & 0\\ 0 & \text{cos }\theta \; & -\text{sin }\theta \;\\ 0 & \text{sin }\theta \; & \text{cos }\theta \; \end{array}\right]$$ (2)

where θ is the rotation angle around x*-*axis. The expression of [*T*] is as follows, while the moving platform rotating around y*-*axis:

$$\left[T_{\text{y}}\right]\text{=}\left[\begin{array}{ccc} \text{cos }\varphi \; & \text{0} & \text{sin }\varphi \;\\ \text{0} & \text{1} & \text{0}\\ -\text{sin }\varphi \; & \text{0} & \text{cos }\varphi \; \end{array}\right]$$ (3)

where φ is the rotation angle around y*-*axis. The expression of [*T*] is as follows while the moving platform rotating around z*-*axis:

$$\left[T_{\text{z}}\right]\text{=}\left[\begin{array}{ccc} \text{cos }\psi \; & -\text{sin }\psi \; & \text{0}\\ \text{sin }\psi \; & \text{cos }\psi \; & \text{0}\\ \text{0} & \text{0} & \text{1} \end{array}\right]$$ (4)

where ψ is the rotation angle around z*-*axis.

The coordinates of each point at the initial state are expressed as follows:

$$\begin{array}{l} B_{1}={\left\{\begin{array}{ccc} 409 & -416.9 & 0 \end{array}\right\}}^{\text{T}}\\ B_{2}={\left\{\begin{array}{ccc} 565.5 & -145.7 & 0 \end{array}\right\}}^{\text{T}}\\ B_{3}={\left\{\begin{array}{ccc} 156.6 & 562.6 & 0 \end{array}\right\}}^{\text{T}}\\ B_{4}={\left\{\begin{array}{ccc} -156.6 & 562.6 & 0 \end{array}\right\}}^{\text{T}}\\ B_{5}={\left\{\begin{array}{ccc} -565.5 & -145.7 & 0 \end{array}\right\}}^{\text{T}}\\ B_{6}={\left\{\begin{array}{ccc} -409 & -416.9 & 0 \end{array}\right\}}^{\text{T}} \end{array}$$ (5)

$$\begin{array}{l} b_{\text{1}}={\left\{\begin{array}{ccc} 143.2 & -514.5 & 600 \end{array}\right\}}^{\text{T}}\\ b_{2}={\left\{\begin{array}{ccc} 517.1 & 133.3 & 600 \end{array}\right\}}^{\text{T}}\\ b_{3}={\left\{\begin{array}{ccc} 374.0 & 381.2 & 600 \end{array}\right\}}^{\text{T}}\\ b_{4}={\left\{\begin{array}{ccc} -374.0 & 381.2 & 600 \end{array}\right\}}^{\text{T}}\\ b_{5}={\left\{\begin{array}{ccc} -517.1 & 133.3 & 600 \end{array}\right\}}^{\text{T}}\\ b_{6}={\left\{\begin{array}{ccc} -143.2 & -514.5 & 600 \end{array}\right\}}^{\text{T}} \end{array}$$ (6)

Accordingly, the lengths of the limbs at the initial state are listed as follows: *l*_1_ = *l*_6_ = 663.5 mm, *l*_2_ = *l*_5_ = 663.5 mm, *l*_3_ = *l*_4_ = 663.5 mm. Furthermore, the freedom analysis of this parallel mechanism is based on the modified G-K formula (Huang et al. 2009):

$$M\text{=}d\left(n-g-1\right)+\sum_{i=1}^{g}f_{i}+\;\upsilon \;$$ (7)

where *M* is the df of this parallel mechanism, *d* represents the order number of the parallel mechanism, *g* is the kinematic pair number of the parallel mechanism, *f*_*i*_ represents the df of the *i*th kinematic pair, and υ is the redundant constraint number of the parallel mechanism. In this parallel mechanism, *d* = 6, *n* = 8, *g* = 9, $\sum_{i=1}^{g}f_{i}=18$, υ = 0. Thus, the freedom of this parallel mechanism is 6.

**Fig. 4.**
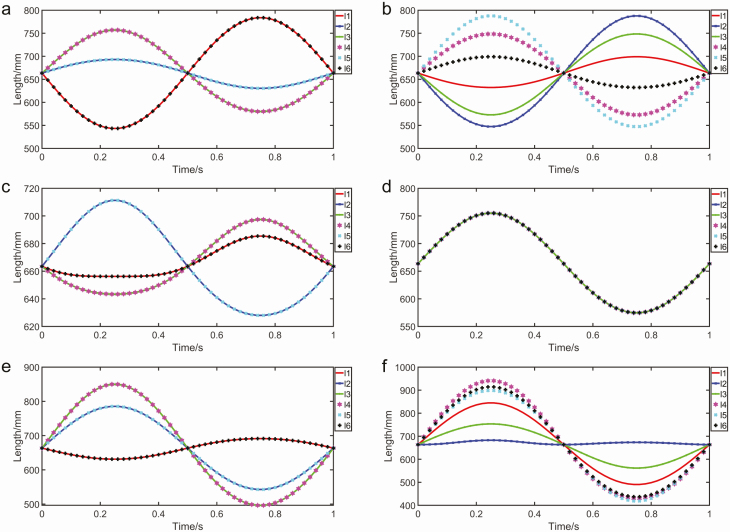
The size changes of the limbs in the simulation of the kinematic analysis of the parallel mechanism. (a) The size changes of the limbs while the moving platform rotating 15 degrees around the x-axis in the sine law. (b) The size changes of the limbs while the moving platform rotating 15 degrees around the y-axis in the sine law. (c) The size changes of the limbs while the moving platform moving 100 mm along the y-axis in the sine law. (d) The size changes of the limbs while the moving platform moving 100 mm along the z-axis in the sine law. (e) The size changes of the limbs while the moving platform rotating 15 degrees around the x-axis and moving 100 mm along the z-axis in the sine law meanwhile. (f) The size changes of the limbs while the moving platform rotating 15 degrees around the x-axis, rotating 15 degrees around the y-axis and moving 100 mm along the z-axis in the sine law at the same time. l, limb.

As shown in the Fig. 4a–f, they are the simulation results through MATLAB based on the abdominal motions. In practice, it is the change in limb length that determines the dexterity of the moving platform. They demonstrate the variation of the limbs length and the changes of the limbs while the moving platform accomplishing different movements. It can be seen from the simulation results that the moving platform can implement a variety of transformations by changing the length of the limbs. This parallel mechanism can simulate all of the intersegmental motions of honey bee abdomen on account of the moving platform has 6 degrees.

## Discussion

**Fig. 5.**
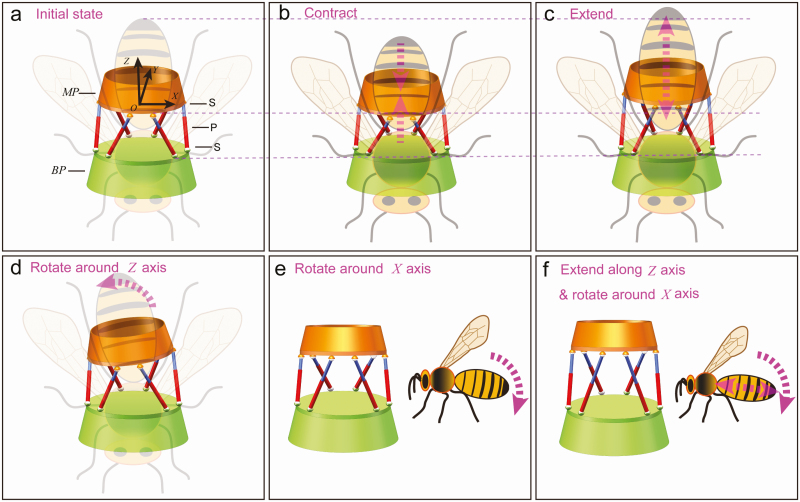
The qualitative kinematic analysis of the parallel mechanism based on the motion of honey bee abdomen. (a) The initial state of the parallel mechanism and honey bee abdomen; MP, moving platform; BP, base platform; S, spherical pair; P, prismatic pair. (b) The distance between the moving platform and base platform gets shorter. (c) The distance between the moving platform and base platform gets longer. (d) The moving platform rotates around z-axis. (e) The moving platform rotates around x-axis. (f) The moving platform extends along z-axis and rotates around x-axis.

For the purpose of verifying the correctness of the physical model, the qualitative kinematic analysis of this mechanism is carried out. As demonstrated in Fig. 5a, it is the initial state of the parallel mechanism and honey bee abdomen. The coordinate system is located on the moving platform. As shown in Fig. 5b, the honey bee contracts its abdomen and the distance between the cuticle segments gets shorter. The corresponding posture of the parallel mechanism is manifested in the image and each limb shortens the same length. In contrast, the honey bee extends its abdomen and the distance between the cuticles gets longer. The distance between the moving platform and base platform increases (Fig. 5c). As demonstrated in Fig. 5d, the honey bee abdomen can rotate around z-axis. This motion is closely related to the waggle dance and other relevant behavioral activities. Different size changes of the parallel mechanism limbs have taken place to keep pace with the motion of honey bee abdomen. Subsequently, the honey bee abdomen can not only rotate around x-axis (Fig. 5e), but also can rotate around x*-*axis and extend along y-axis meanwhile (Fig. 5f). As for the parallel mechanism, the change along the z-axis is consistent with the change of the abdomen along the y-axis. The motions of honey bee abdomen correspond to the related behavioral activities such as waggle dance, flight attitude adjustment, and sting.

A conclusion can be drawn from the above analysis that the parallel mechanism can complete all intersegmental motion tasks successfully. Furthermore, this parallel mechanism may be also applicable to illustrate the deformation mechanism of dragonflies (Mill 1964), butterflies (Wasserthal and Wasserthal 1980) and drosophilae (Currie and Bate 1991), since these three insects have similar intersegmental structures. This morphing structure provides an inspiration for the design of bionic structure such as nose cone of aerospace vehicle (Zhao et al. 2017b, Zhang et al. 2019, Liang et al. 2020).
